# Daily consumption of a synbiotic yogurt decreases energy intake but does not improve gastrointestinal transit time: a double-blind, randomized, crossover study in healthy adults

**DOI:** 10.1186/1475-2891-12-87

**Published:** 2013-06-20

**Authors:** Hilary M F Tulk, Diane C Blonski, Lauren A Murch, Alison M Duncan, Amanda J Wright

**Affiliations:** 1 Department of Human Health and Nutritional Sciences, University of Guelph, Guelph, Ontario N1G 2W1, Canada

**Keywords:** Synbiotics, Yogurt, Gastrointestinal transit, Energy intake

## Abstract

**Objective:**

Probiotic and synbiotic products are widely marketed to healthy individuals, although potential benefits for these individuals are rarely studied. This study investigated the effect of daily consumption of a synbiotic yogurt on gastrointestinal (GI) function in a sample of healthy adults.

**Subjects/Methods:**

In a randomized crossover double-blind study, 65 healthy adults consumed 200 g/day of yogurt with (synbiotic) or without (control) added probiotics (*Bifidobacterium lactis* Bb12, *Lactobacillus acidophilus* La5*, Lactobacillus casei* CRL431) and 4 g inulin for two 15-day treatment periods, each preceded by a 6-week washout period. GI transit time (GTT), duration of colour (DOC), GI symptoms and dietary intake were assessed and analyzed using repeated measures ANOVA, including PRE-treatment GTT as a covariate. Participants were grouped as short GTT (STT, n = 50, ≤32.7 h) or long GTT (LTT, n = 15, >32.7 h) based on their PRE-treatment GTT assessment.

**Results:**

POST-treatment GTT and DOC were not different between synbiotic and control, and did not change from PRE-treatment, within the STT or LTT groups. There were no changes in GI symptom ratings, indicating that both yogurts were well tolerated. In STT, energy, fat and protein intakes were decreased from baseline with synbiotic (p = 0.055, p = 0.059 and p = 0.005, respectively) and dietary fibre intake was higher POST-treatment with synbiotic versus control (p = 0.0002). In LTT, decreases in energy and fat intakes with synbiotic were not significant (p = 0.14 and p = 0.18, respectively) and there were no differences in dietary fibre intake.

**Conclusion:**

Consuming 200 g/day of synbiotic yogurt did not significantly alter GTT in healthy adults, but was well tolerated and helped to reduce overall energy intake.

## Introduction

It is well established that the gastrointestinal (GI) microbiota impacts overall health and that the quantities and types of bacteria composing the microbiota can be optimized by dietary factors, particularly prebiotics and probiotics [1]. Consequently, consumers are turning to probiotic-containing foods for relief of specific conditions or to improve overall health [2]. However, evidence to support health claims for probiotics and synbiotics (containing prebiotics and probiotics) in the general population, the main group to whom these products are marketed, is limited [2,3]. The efficacy of synbiotics can vary depending on the probiotic strain and the food matrix can affect probiotic survival and potential health benefits [4,5]. Strain- and product-specific research to substantiate the efficacy of probiotics in improving or maintaining GI health in healthy consumers is required [2-4,6].

Probiotics have traditionally been used to treat GI diseases and disturbances, including lactose intolerance, rotavirus and antibiotic associated diarrhea, inflammatory bowel disease and constipation [3,7,8]. Consumption of fermented dairy products containing *Bifidobacterium lactis* DN-173010 (≥10^8^ CFU/g) has been shown to improve bowel movement frequency and consistency in women with constipation [9], to decrease initially long GI transit times (GTT) in healthy women [10], men [11] and older adults [12,13], and to improve overall GI well-being and digestive symptoms in healthy women [14]. Intestinal transit time tended to decrease (p = 0.055) in healthy Japanese women with slow transit times (> 40 hours) whom consumed 170 g/day yogurt containing 10^8^ CFU/g of the same probiotic strain [15].

A growing body of work exists with respect to potential GI health benefits of dairy products containing various probiotic strains [16-18] including B*ifidobacterium lactis* Bb12 and *Lactobacillus acidophilus* La5 [19-21]. Savard et al. [2011] showed that these probiotics consumed for 4 weeks from a commercial yogurt survived GI transit and led to increases and decreases, respectively, in the presence of beneficial and potentially pathogenic gut bacteria. Consumption of yogurts containing Bb12, in combination with *Lactobacillus acidophilus* La5, has also been shown to suppress H. pylori infections [22], to improve antioxidant status in participants with type 2 diabetes [23], and to maintain serum insulin levels in pregnant women [24].

Prebiotics are non-digestible carbohydrates that selectively stimulate the growth of beneficial bacteria in the GI tract [25]. Fructooligosaccharides, in particular inulin, are commonly added to foods in concentrations from 0.6-2.1 g/100 g [1]. Inulin is a soluble prebiotic fibre that has been shown to increase fecal *Bifidobacteria* spp. levels [26], to increase stool bulk and frequency when initially low [27], and to exert a mild laxative effect in elderly people with constipation [28]. As well, 16 g/day of inulin and oligosaccharides decreased subjective hunger scores and increased concentrations of plasma gut peptides that regulate food intake, suggesting a satiety effect in healthy adults [29]. However, research to establish the relevance of satiety-promoting foods, in terms of their long-term ability to modulate food intake and to beneficially impact body weight, is required. Also, while there is rationale for enriching foods with prebiotics, some studies have associated prebiotic consumption, including inulin, with mild GI side effects [29-31]. Since functional foods that modulate energy and increase fibre intake may be an important strategy in addressing overweight, obesity and their associated conditions [29,31,32], research addressing the tolerance of prebiotics in the general population is warranted.

Consumption of dairy products, including yogurt, by North American and European consumers is considerable and dairy products currently lead the probiotic market [33]. This is, in part, due to the healthy perceptions and familiarity consumers have with fermented dairy products containing live bacteria [5] and to the fact that live strains of probiotics can be added to these products relatively easily [34]. Yogurt, in particular, is considered to be a suitable synbiotic food matrix [34] since it is intrinsically thickened and its texture should remain acceptable if prebiotic fibre-addition induces further thickening [35]. Addition of a prebiotic to dairy products containing probiotics has also been shown to improve the survival of probiotic bacteria during their shelf-life [36,37]. Therefore, synbiotics are an exciting area of growth for the dairy sector [1]. More broadly, a growing body of evidence supports a role for various synbiotic supplements and foods in improving GI disturbances [14,38-42].

There is also evidence that fibre-enriched yogurts can suppress short-term appetite and may be effective in controlling food intake [43,44]. For example, yogurt was found to have greater satiating effects than isocaloric fruit-based or dairy fruit beverages [45] and the effect was enhanced when yogurt was enriched with 6 g of inulin [46]. Therefore, optimizing the microbial population of the GI tract through the consumption of synbiotics could be a strategy in preventing obesity, in addition to other chronic diseases such as inflammatory bowel disorders, and some cancers [3,29,32,47]. This study investigated whether daily consumption of a synbiotic yogurt improves GI function in a sample of healthy Canadian adults. GTT, GI symptoms and food intake were compared between participants with short or long baseline GTT following consumption of a synbiotic yogurt or a control product without added probiotics and inulin.

## Methods

### Study design

This study was conducted at the Human Nutraceutical Research Unit (HNRU) in the Department of Human Health and Nutritional Sciences at the University of Guelph and was approved by the University’s Human Research Ethics Board. A randomized crossover double-blind design was utilized and consisted of two 15-day treatment periods, each preceded by a 6-week washout period.

### Participants

Healthy males and females (18 to 65 y, BMI 18–35 kg/m^2^) were recruited from the local community and pre-screened using a phone/email questionnaire. Individuals were excluded if they had food allergies or intolerances, GI disorders or disease or if they regularly used medications (except hormonal contraceptives), natural health products or dietary supplements, or had used antibiotic medication <3 months prior to the study. Individuals who smoked or were elite or varsity athletes and/or training for a major athletic event, and females who were pregnant, lactating or not practicing birth control, were also excluded. All participants provided written informed consent and attended an orientation session prior to the study.

### Determination of gastrointestinal transit time

GTT was measured using capsules containing 0.25 g of carmine red (Sensient Colours Canada Ltd., Kingston, ON) and 0.15 g of carbon black (Castleguard Health Services, Paris, ON). Carmine red is a non-digestible food colourant that produces a distinct red colour in the feces [48,49] which is enhanced by the presence of carbon black [13].

Participants consumed 2.0 g carmine red and 0.5 g carbon black with breakfast on two mornings, separated by 72 h, for duplicate determination of GTT. They recorded the date and time of capsule consumption and the date, time and colour of all subsequent bowel movements until both doses of colour had passed. GTT was defined as the length of time (h) between capsule consumption and appearance of red colour in the feces and is reported as the average time for both doses to appear in the feces. The duration of red colour (DOC) in the feces was also calculated, as a secondary indicator of GI transit. DOC was defined as the length of time (h) between capsule consumption and the last appearance of red colour in the feces and is reported as the average time for both doses to disappear from the feces. DOC relates to the amount of time the bowel mucosa is exposed to digesta, including possible carcinogens [49].

At the start of the 6-week washout period, a baseline GTT assessment was completed to familiarize participants with the GTT assessment protocol and to screen for participants with difficulty detecting the red colour in their feces. Baseline GTT data was used for preliminary examination of the distribution of GTT within participants. GTT assessments were completed PRE-treatment (commencing 3 days prior to Day 1) and POST-treatment (commencing on Day 13) during each 15-day treatment period.

### Study treatments

Participants were instructed to maintain their usual lifestyle and dietary habits with specific instructions to avoid yogurt products and foods with added prebiotics or probiotics, with the exception of the study treatments. The study treatments were industrially manufactured vanilla flavoured yogurts with (symbiotic) and without (control) the addition of *Bifidobacterium lactis* Bb12 (≥10^7^ CFU/g), *Lactobacillus acidophilus* La5 (≥10^7^ CFU/g)*, Lactobacillus casei* CRL431 (≥10^7^ CFU/g) and 2 g inulin (average degree of polymerization ≥ 10, Beneo Raftiline) per 100 g serving. Probiotic counts ≥ 10^9^ CFU per 100 g serving were verified by pour plate count methods throughout the treatment period. Both yogurts were produced using identical starter cultures and milk ingredients and were manufactured for each treatment period separately so that the products were consumed at the same point in their shelf life. Treatments were packaged as 100 g servings in opaque white cups, stored under refrigeration and provided to participants immediately prior to the start of each treatment period. Per 100 g serving, the yogurts contained 100 kcal, 3.0 g protein, 18.0 g carbohydrate and 3.0 g fat. Participants were instructed to consume two 100 g servings of yogurt per day during each treatment period and to keep the yogurt refrigerated. Participants recorded the date, time, and other details about their yogurt consumption in a daily study diary.

### Baseline anthropometric data

Five days prior to the first treatment period, participants reported to the HNRU after a 12–14 h overnight fast. Height was measured to the nearest 0.1 cm using a stadiometer (SECA Portable Stadiometer 214, Hanover, MD, USA) and body weight was measured to the nearest 0.1 kg using a calibrated digital scale (Acculab SV-100, Edgewood, NY, USA). Body fat (%) was determined by bioelectric impedance analysis (BodyStat 1500™, BodyStat Ltd., Douglas, IOM).

### Gastrointestinal symptoms

A modified version of the GI symptom rating scale (GSRS) [50] was used to evaluate changes in perceived GI symptoms at Days 1, 7 and 15 of each treatment period. The modified GSRS included 5 questions pertaining to abdominal discomfort, stomach grumbling, bloating, belching, and flatulence, each scored as 0 for ‘no symptoms’ to 3 for ‘extreme symptoms’, and one question each related to consistency and frequency of bowel movements scored as 0 to 4, where 0 and 4 indicated opposite extremes and 2 indicated normal consistency or frequency.

### Dietary intakes

Three-day food records were collected at the start of the first 6-week washout period (baseline) and on Days 13–15 of each treatment period (POST-treatment). Participants were provided with detailed instructions for food record completion. Food records were reviewed with a study coordinator upon submission, analyzed using The Food Processor® SQL version 10.3.0.0 (ESHA Research Salem, OR, USA) and 3-day means for energy, fat, protein, carbohydrate and dietary fibre were calculated.

### Data and statistical analysis

**Figure 1:**
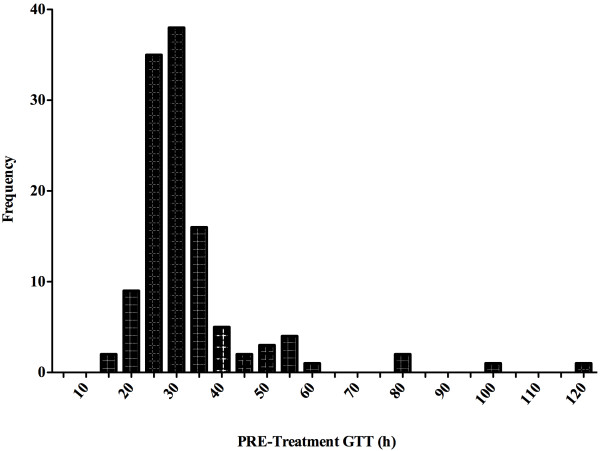
**Histogram depicting the distribution of PRE-treatment GTT **^**1**^**.** Abbreviations: GTT, gastrointestinal transit time. ^1^ Distribution of PRE-treatment GTT values, measured in duplicate in two separate GTT assessments, for each participant (n = 65).

The intra-individual variation in PRE-treatment GTT assessment data was examined for each participant. Participant data was excluded from statistical analysis if the PRE-treatment GTT was significantly different between synbiotic and control or if the coefficient of variation for PRE-treatment GTT was ≥40%. Participants were grouped as short GTT (STT) or long GTT (LTT) based on [13] and [12]. The distribution of PRE-treatment GTT was examined using a histogram to determine a cut-off point for grouping of participants as STT or LTT (Figure 1).

GTT was analyzed for effect of treatment within STT and LTT groups, both between treatments (using repeated measures ANOVA on POST-treatment values and controlling for participant, treatment and treatment order and including PRE-treatment values as a covariate), and within treatments (using a paired *t*-test on PRE- and POST-treatment values within a treatment). Baseline anthropometric data were compared between STT and LTT groups using an unpaired *t*-test. GSRS scores were analyzed within STT and LTT groups for effect of time (i.e. Days 1 vs. 7 vs. 15) within treatments and for the effect of treatment within study days, using Chi squared analysis. Dietary intake data was analyzed within STT and LTT groups for effect of time (i.e. baseline vs. POST-treatment) within treatments and for effect of treatment (on POST-treatment values), both using unpaired t-tests. All statistical analyses were performed using SAS version 9.1 (SAS Institute, Inc., Cary, NC) and a significance level of p <0.05.

## Results

### Participant flow and GTT grouping

Of the 300 potential participants screened by phone or email, 158 attended a screening appointment and 127 were eligible for the study, assigned participant numbers and invited to complete a baseline GTT assessment. 111 participants completed the baseline GTT assessment. Of these, 28 subsequently dropped out or were excluded from the study due to scheduling conflicts (n = 9), use of antibiotics or other medications (n = 3), difficulty swallowing the capsules (n = 2), GI upset or diarrhea (n = 6), or not wishing to continue (n = 8). Therefore, 83 participants entered the first treatment period, during which 4 participants discontinued their participation due to illness (n = 2), scheduling conflicts (n = 1), and antibiotic use (n = 1). After completion of the first treatment period, 10 participants were excluded from the study due to GI upset (i.e. diarrhea) during the GTT assessments (n = 5), non-compliance to study protocol (n = 1), commencement of antibiotics or prescription drugs (n = 2) or scheduling conflicts (n = 2). After analysis of the PRE-treatment GTT for intra-individual variation ≥40%, 4 more participants were excluded. The distribution of PRE-treatment GTT for each treatment period was examined for the 65 participants whose data was included in the statistical analysis. GTT data was divided at the 75th percentile with participants grouped as either short GTT (STT; n = 50, ≤32.7 h) or long GTT (LTT; n = 15, >32.7 h). Sample size was estimated at 41 participants for the STT group and 17 participants for the LTT group using effect sizes (i.e. time reductions) of 4 hours for STT and 10 hours for LTT, standard deviation estimates of 5 for STT and 8 for LTT, a power of 95%, an alpha of 0.05 and 2-sided testing.

### Participant characteristics

At baseline, participants had a mean age of 28.6 y, body weight of 72.1 kg, height of 171.2 cm, BMI of 24.4 kg/m^2^ and body fat of 23.0%. There were no significant differences in participant characteristics between the STT and LTT groups (Table 1). The STT group consisted of 23 men and 27 women, whereas the LTT group consisted predominantly of women (n = 15) versus men (n = 3).

**Table 1:** **Baseline characteristics for all participants and for STT and LTT groups**^**1**^

	**All participants**	**STT**	**LTT**
	**(n = 65)**	**(n = 50)**	**(n = 15)**
Males	n = 26	n = 23	n = 3
Females	n = 39	n = 27	n = 12
Age (y)	28.6 ± 11.3	28.0 ± 10.6	30.7 ± 13.2
Height (cm)	171.2 ± 8.9	171.0 ± 8.5	171.9 ± 8.9
Body weight (kg)	72.1 ± 15.3	72.4 ± 14.1	71.0 ± 18.5
Body mass index (kg/m^2^)	24.4 ± 4.0	24.6 ± 3.5	23.8 ± 4.6
Body fat (%)	23.0 ± 8.9	22.4 ± 8.5	25.9 ± 9.3

^1^ Mean ± SD.

### Gastrointestinal transit time assessments

There were no significant differences in POST-treatment GTT or DOC between the synbiotic and control yogurts within either the STT or the LTT groups. Similarly, within the STT and LTT groups there were no significant differences between PRE-treatment and POST-treatment GTT or DOC, within either the synbiotic or control yogurts (Table 2).

**Table 2:** **Average GTT (h) and DOC (h) for participants with STT and LTT PRE- and POST-treatment**^**1**^

	**All participants**		**STT (n = 50)**		**LTT (n = 15)**	
	**Synbiotic**	**Control**	**Synbiotic**	**Control**	**Synbiotic**	**Control**
GTT PRE	31.0 ± 16.6	29.8 ± 12.7	26.1 ± 4.1	24.4 ± 4.4	51.7 ± 28.7	47.8 ± 14.8
GTT POST	30.6 ± 18.3	31.1 ± 15.4	26.3 ± 8.2	26.7 ± 10.4	48.8 ± 33.9	45.7 ± 20.3
DOC PRE	50.4 ± 20.1	52.1 ± 22.9	44.6 ± 15.1	45.4 ± 13.4	71.4 ± 22.1	73.6 ± 32.9
DOC POST	52.8 ± 28.3	51.5 ± 22.0	44.7 ± 14.9	46.3 ± 15.9	82.0 ± 43.5	68.2 ± 30.1

Abbreviations: *GTT* gastrointestinal transit time, *DOC* duration of colour, *STT* short gastrointestinal transit time, *LTT* long gastrointestinal transit time, *PRE* pre-treatment, *POST* post-treatment.

^1^ Mean ± SD.

### Gastrointestinal symptoms

**Figure 2:**
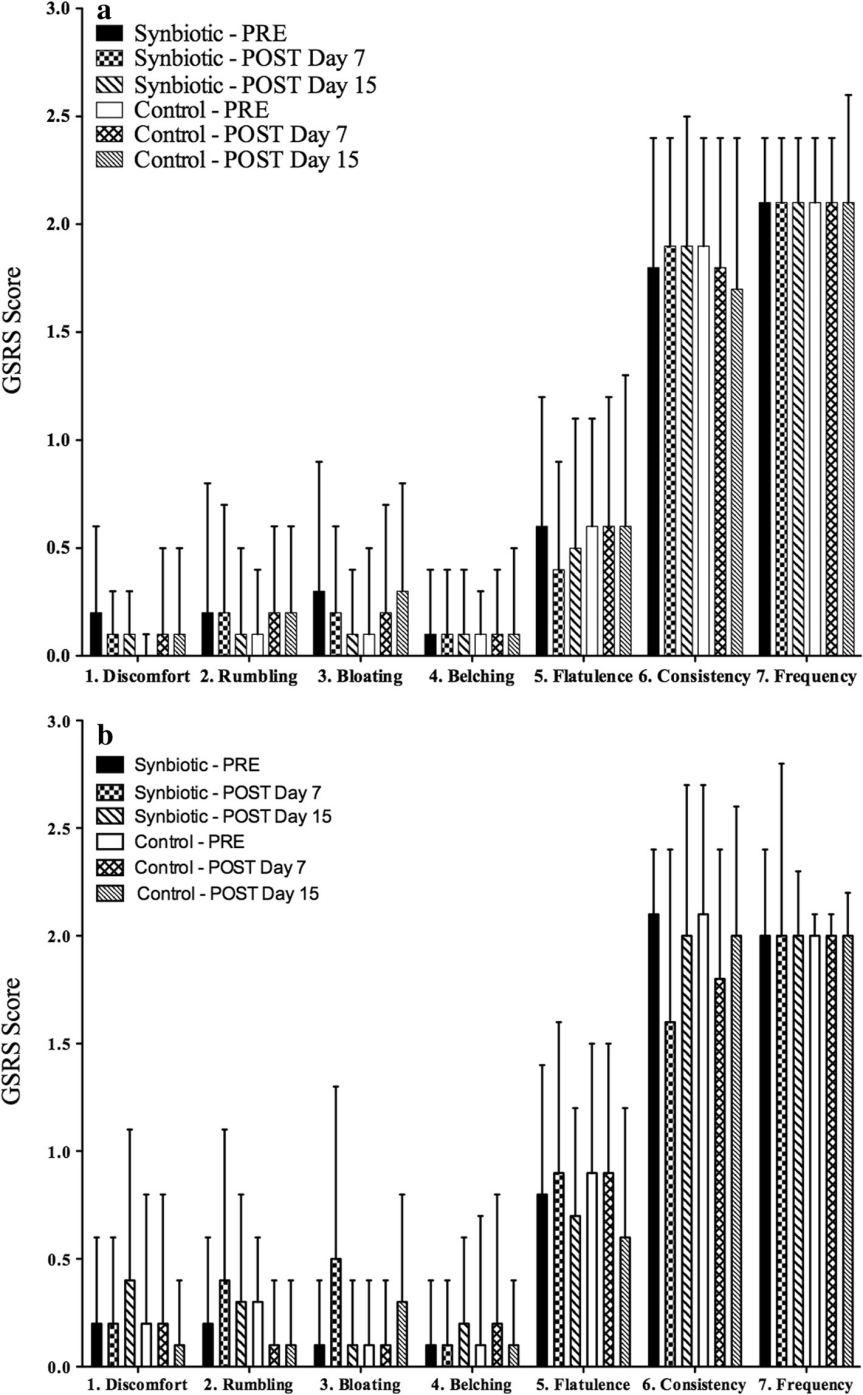
**STT (a) and LTT (b) GSRS scores for PRE, POST 7 and POST 15 days of treatment **^**1,2,3,**^**.** Abbreviations: GSRS, gastrointestinal symptom rating scale; STT, short gastrointestinal transit time; LTT, long gastrointestinal transit time; PRE, pre-treatment; POST, post-treatment. ^1^ Average GSRS scores. ^2^ Mean ± SD. ^3^ Questions 1–5 scored as 0 for ‘no symptoms’ to 3 for ‘extreme symptoms’ and questions 6 & 7 scored as 0 to 4, where 0 and 4 indicated opposite extremes and 2 indicated normal bowel movement consistency or frequency.

Both the synbiotic and control yogurts were well tolerated, based on participant ratings of abdominal discomfort, abdominal rumbling, abdominal bloating, belching, flatulence, bowel movement consistency and bowel movement frequency assessed by GSRS scores, within or between treatments, within both the STT or LTT groups (Figure 2).

### Dietary intake

Dietary intake data is presented in Table 3. In the STT group, energy, fat and protein intakes decreased from baseline following consumption of the synbiotic yogurt (p = 0.055, p = 0.059 and p = 0.005, respectively) and carbohydrate intake decreased from baseline following consumption of the control yogurt (p = 0.008). In the LTT group, energy and fat decreased from baseline following consumption of the synbiotic yogurt, but these changes did not reach statistical significance (p = 0.14 and p = 0.18, respectively). In the STT group, dietary fibre intake did not significantly change with consumption of the synbiotic, but decreased from baseline with consumption of the control (p = 0.0002) and was significantly higher POST-treatment with the synbiotic versus control treatment (p = 0.0002). In the LTT group, there was no significant change in dietary fibre intake from baseline with the synbiotic (p = 0.25) or control (p = 0.24) or POST-treatment differences between the synbiotic and control (p = 0.58).

**Table 3:** **Average energy and macronutrient intakes at baseline and POST treatment **^**1,2,3,4**^

	**STT**			**LTT**		
	**Baseline **^**3**^	**Synbiotic **^**5**^	**Control **^**5**^	**Baseline **^**3**^	**Synbiotic **^**5**^	**Control **^**5**^
Energy (kcal)	2498 ± 613	2274 ± 618 ^6^	2325 ± 568	2526 ± 730	2253 ± 418	2470 ± 478
Protein (g)	97.2 ± 30.9 ^a^	85.3 ± 26.7 ^b^	94.7 ± 32.2 ^a,b^	93.8 ± 21.7	85.1 ± 18.6	90.7 ± 20.2
Fat (g)	90.2 ± 42.2	80.0 ± 28.8 ^7^	88.4 ± 30.9	89.5 ± 35.6	75.2 ± 20.6	95.8 ± 31.1
Total Carbohydrate (g)	324.1 ± 88.4 ^a^	304.9 ± 97.4 ^a,b^	285.9 ± 68.6 ^b^	341.4 ± 125.9	319.4 ± 65.3	316.8 ± 63.6
Dietary Fibre (g)	28.1 ± 12.3 ^a^	26.2 ± 9.6 ^a^	20.9 ± 8.2 ^b^	29.3 ± 12.8	27.6 ± 7.6	25.3 ± 7.9

Abbreviations: *STT* short gastrointestinal transit time, *LTT* long gastrointestinal transit time.

^1^ Data are mean ± SD.

^2^ Means in a row with different superscript letters a & b indicate a difference between treatments or from baseline within STT or LTT (p < 0.05).

^3^ Based on 3-day food records completed at commencement of first washout period.

^4^ Data includes contributions from the investigational yogurts.

^5^ Based on 3-day food records completed on Days 13, 14 and 15 of each treatment period.

^6^ p = 0.055 between Baseline and Synbiotic.

^7^ p = 0.059 between Baseline and Synbiotic.

## Discussion

Although claims of improved GI function are widely implied in the marketing of probiotic and synbiotic dairy products, the roles of these products in improving GI function in healthy individuals requires further investigation. Furthermore, there are few reports of typical GTT in healthy Canadians. To our knowledge, this study is the first to examine the effects of a synbiotic yogurt on measures of GI function and dietary intake in healthy Canadian adults.

The average PRE-treatment GTT in this study was in the range of 30 h, based on all participants. This is slightly lower than the 41.0 ± 18.9 h reported for a sample of healthy Canadian adults consuming their habitual diet and determined using fecal x-ray monitoring for passage of radiopaque pellets [51]. Consuming a synbiotic yogurt did not change GTT or DOC from baseline and was not different from the control yogurt, for either the STT or LTT groups. Past research demonstrates that DOC can vary even among individuals with the same frequency of bowel movements, particularly in those with longer transit times [49]. In this study, high variability in DOC was observed in both STT and LTT PRE- and POST-treatment.

Our STT and LTT groups had baseline GTT 25.2 ± 0.4 h and 49.5 ± 4.3 h, respectively. In a recent study, GTT, measured using the dye marker technique, was lowered in women, especially those with functional constipation (transit time ≥48 h), whom consumed two 125 g servings of synbiotic yogurt containing 0.625 g inulin and oligofructose with *Bifidobacterium lactis* Bb12 (10^9^-10^10^ CFU/g) and *Lactobacillus casei* CRL (1 × 10^9^- 6 × 10^9^ CFU/g) daily for 15 days [20]. Similarly, consumption of 2 daily servings for 14 days of a commercial yogurt containing *B. animalis* DN-173 010 (10^8^ CFU/g) and fructoligosaccharide by women with and without functional constipation improved measures of bowel evacuation [41]. Sairanen (2007) used radio-opaque marker detection in feces and, similar to the present study, found no improvements in transit time in healthy men and women whom, for 3 weeks, consumed 600 mL fermented milk with 12 g inulin and probiotics (*Bifidobacterium longum* BB536, *Bifidobacterium* spp. 420 and *Lactobacillus acidophilus* 145) versus fermented milk with the probiotics or a fermented milk control. In that study, average baseline transit times for the treatment groups ranged from 48.6 ± 25.6 to 53.5 ± 22.4 h.

There is consistent evidence to support the benefits of probiotic-enriched dairy products in improving GTT, even with relatively short nutrition interventions, if GTT is initially slow [9,10,12,13]. However, in healthy individuals, there is minimal room for improvement in GTT, the measurements of which can vary from 20 to 30%, even with strict dietary controls [47,51,52]. Therefore, the relatively short initial GTT of the healthy and relatively young (28.6 ± 11.3 years) participants in the present study may help to explain the finding of no improvements in GTT. Of note, the physiological significance of small changes in GTT potentially induced in healthy individuals has not been confirmed. Conceivably, it would be undesirable for a product to excessively shorten a GTT such that it had laxative effects [30].

Differences between the results of the current study and those previously reported may be related to differences in probiotic strain and dosage, prebiotic type and concentration, and food matrix. The use of different techniques to determine GI transit may also explain apparent inconsistencies between studies. In the present study, use of the dye marker technique, while relatively non-invasive, inexpensive and conducive to studies with a relatively large number of participants, relies on participant self-reports of timing for dye ingestion and fecal appearance. It also cannot reveal transit times within specific segments of the GI-tract, where differences may exist [53].

Probiotic-containing foods are generally well tolerated, with minimal adverse side effects or undesirable GI symptoms [10]. However, the tolerability of products containing both probiotics and prebiotics is understudied and expected to be product-specific. In the current study, supplementing the daily diet with probiotic- and inulin-enriched yogurt was well tolerated by healthy adults. Consumption of the same probiotic strains in yogurt also did not result in incidence of adverse GI symptoms beyond that observed with the placebo yogurt [21]. Similarly consumption of 10^8^ to 10^11^ CFU of *Bifidobacterium animalis* and *Lactobacillus paracasei* per day did not induce adverse effects and was generally well tolerated in healthy adults [19]. In comparison to a milk control, milk fermented with *Bifidobacterium lactis* DN173010 was found to improve overall GI symptoms in women who reported minor digestive symptoms at baseline, mainly due to improvements in gas-related symptoms [14]. In contrast, intestinal discomfort from gas production has been implicated as a side effect of prebiotic consumption [1]. One study found that healthy adults experienced a significant increase in GI symptoms when consuming probiotic fermented milk with 12 g of inulin per day (4 g per 200 ml serving) compared to the probiotic and control fermented milks [30]. However, inulin consumed in doses of 5 and 10 g is generally well tolerated [31]. Healthy adults whom consumed a supplement containing probiotics which included Bb12 and La5, as in the present study, reported beneficial effects on bowel habits [38]. A prebiotic dose of 5 g/day is reportedly sufficient to elicit a positive effect on the GI microbial population [1]. The current study delivered 4 g of inulin per day (2 g per 100 g serving) and was well tolerated by healthy adults. Collectively, these results suggest that consumption of fermented dairy products, including or in conjunction with, a moderate amount of inulin (≤10 g per day) does not induce undesirable GI symptoms in healthy adults [34,54].

Analysis of dietary intake data revealed that energy intake was decreased in the STT and trended towards a decrease in the LTT during consumption of the synbiotic yogurt. The consumption of prebiotic fibres, including inulin-type fructans, can impact appetite-related endpoints and may play a role in appetite regulation and energy intake [55,56]. Reductions in daily energy intake by 100 kcal, similar to the 120 kcal observed in this study can impact energy balance sufficiently to prevent weight gain [57]. This indicates that regular consumption of the synbiotic could translate into measurable and beneficial changes in dietary intake in free-living, healthy adults. In the LTT group, no differences in dietary fiber intake were observed between baseline and POST-treatment. However, in the STT group dietary fibre intake significantly decreased in the control group, but was maintained with consumption of the synbiotic yogurt. This indicates that consuming two daily servings of a synbiotic dairy product with 2 g inulin may reduce energy intake, while contributing dietary fibre. Since the average dietary fibre intake of adults in Canada and the United States fails to meet Adequate Intakes of 38 g/day for males and 25 g/day for females [58], food-based strategies that support this aim are warranted. The current study is unique in that dietary intake was measured in healthy Canadian adults after 15 days of supplementing their diets with a synbiotic yogurt, whereas other studies have measured short-term hunger and satiety in laboratory settings [43-45]. Limitations of the study include the reliance on self-reported nutrient intake by participants and the fact that the significant reduction in energy intake was observed in the STT, but not the LTT group.

## Conclusion

Consumption of a synbiotic yogurt containing *Bifidobacterium lactis* Bb12*, Lactobacillus acidophilus La5*, *Lactobacillus casei* CRL431 and inulin for 15 days did not significantly alter measures of GTT in a sample of 65 healthy Canadian adults. However, it was well tolerated according to GI symptom scores and its consumption was associated with reductions in energy intake. The study adds to the limited literature investigating the potential of synbiotic products to modulate GI function in healthy adults.

## Abbreviations

GI: Gastrointestinal; GTT: Gastrointestinal transit time; DOC: Duration of colour; STT: Short transit time; LTT: Long transit time; HNRU: Human Nutraceutical Research Unit.

## Competing interests

The authors were solely responsible for study design, data collection, analysis, interpretation, manuscript preparation, and decision to publish the results.

## Authors’ contributions

HMFT & DCB jointly contributed to preparation of the manuscript, data collection and analysis. LAM contributed to data collection and entry. AJW and AMD jointly led the study as co-principal investigators and supervised all personnel. All authors read and approved the final manuscript.
